# Association between physical health and physical activity behaviors for children aged 3–6 years in kindergarten: A cross-sectional study from China

**DOI:** 10.1371/journal.pone.0278341

**Published:** 2022-12-15

**Authors:** Xin Xiong, Weinan Zhang, Yaqi An, Yunchen Meng, Haifeng Li, Zhiping Zhen, Jian Sun

**Affiliations:** 1 College of P.E and Sports, Beijing Normal University, Beijing, China; 2 Guangzhou Sport University, GuangZhou, China; University of Study of Bari Aldo Moro, ITALY

## Abstract

**Purpose:**

To explore the association between the physical health (PH) and physical activity behavior (PAB) of children aged 3 to 6 years, and to provide a basis for the scientific planning of children’s physical activity behavior in kindergarten.

**Methods:**

A total of 18041 children in China aged 3 to 6 years were selected as the research subject. The PH monitoring indicators were measured according to "The National Physical Fitness Measurement Standards Manual" (Preschool Children Version) (NPFMSM), and PAB was reported by teachers. The chi-square test was used to test for differences in PH and PAB across groups, the percentile method was used to rank PAB, correlation analysis was used to analyze the association between components of PH and PAB, and multiple linear regression was used to determine the independent association between PAB and PH.

**Results:**

Participants in this study exhibited poor PH status, and the detection rates of failure were 16.4% for boys and 16.8% for girls, and showed a decreasing trend with increasing age. The grade difference and sex difference in PH components were statistically significant (P<0.01). PAB changes to static activities with increasing age, mainly to static indoor physical activities (SIPAs). The regression effects of the total duration of physical activity (TDPA) and dynamic physical activity (DPA) on PH score and physical fitness (PF) score were significant (P < 0.01) but not statistically significant with static physical activity (SPA) (P>0.05). Dynamic outdoor physical activity (DOPA) is the core factor affecting children’s PH, and is significantly positively correlated with the components of PH.

**Conclusion:**

PAB in kindergarten can improve children’s PH, and reasonable planning of PAB in kindergarten has a targeted effect on PH promotion.

## 1 Introduction

Physical activity is closely related to human health. Adequate and regular physical activity is an important cornerstone of children’s physical and mental health, effectively promoting children’s bone health and cardiometabolic health, improving cardiopulmonary fitness and physical fitness, promoting cognitive function development and helping to control weight and reduce the risk of psychological depression [1]. With the rapid development of modern society, human lifestyles and behavioral habits are undergoing major changes. The sedentary lifestyles of screen viewing, traveling by car and sitting in the office are becoming more common and occurring at a younger age. The World Health Organization (WHO) has ranked sedentary behavior as the fourth highest risk factor for mortality worldwide. According to statistics, approximately 61% to 80% of young children worldwide spend too much time in static activity, and the proportion in China is as high as 88.2%, which is much higher than the global average [2]. Children’s PH problems caused by insufficient physical activity have become an important public health issue facing the current society.

Appropriate PAB is essential to develop good exercise habits in children. However, the influencing factors of PAB are complex, including age, sex, physical activity level and many other aspects, and have greater compliance with the living environment. The home and kindergarten are the main living and learning environments for children aged 3 to 6 years, and both have significant impact on children’s PAB. Kindergarten is the living environment with the longest contact time for children with non-family members, and it is also the main place for children to engage in physical activities. At the same time, this period is the initial stage of children’s school education, which plays an important role in the development of children’s physical activity habits [3]. According to the teaching characteristics of kindergartens, children’s physical activities are mainly organized indoors or outdoors, and are divided into DPA and SPA. Different types of PAB vary greatly in volume, content and mode, which will also have different effects on PH [4]. Previous studies have mostly focused on the interrelationships and intrinsic patterns among physical activity levels, PH and growth during physical activity in children, and have explored the dose-effect of physical activity on PH promotion, including obesity intervention, PF promotion, and noncommunicable disease prevention [5, 6]. Few studies have focused on the association between children’s PAB and health. Related studies have shown that different types of PAB have great differences in activity volume, activity content, and activity mode. Furthermore, different types of PAB will also have different effects on health. Therefore, clarifying the structure and composition of PAB in kindergarten is helpful to explore the reasons why different PABs have different effects on children’s PH.

The purpose of this study was to analyze the relationship between the PH of children aged 3~6 and the PAB in kindergartens, explore the overall effect of PAB on PH, and explore the independent associations between different PAB types and the components of PH, to provide a theoretical basis for the setting and optimization of children’s PAB in kindergartens.

## 2 Methods

### 2.1 Ethics statement

The study was conducted in accordance with the Declaration of Helsinki, and the experimental protocol was approved by the Ethics Committee of the Faculty of Psychology, Beijing Normal University (Project identification code: 201903260036), and all subjects have their written informed consent.

### 2.2 Study design and population

This was a cross-sectional analysis based on baseline measurements. Our cross-sectional survey was conducted between 2018 and 2019 in Guangdong Province, China. To obtain representative samples, a total of 18,365 children aged 3 to 6 years were surveyed in this study using stratified random sampling, which distinguished between boy and girl. Samples with questionnaire response rates and PH indicators less than 85% were excluded, as well as data with test values outside of 3 times the standard deviation (M±3SD) were excluded, and 18,041 participants (9,168 boys and 8,856 girls) were retained for inclusion in the analysis. The inclusion criteria of this study were as follows: (1) preschool children in kindergartens aged 3 to 6 years; (2) healthy children, excluding contraindications of exercise, cardiovascular diseases, neurological or endocrine diseases; and (3) informed consent of the participant’s parents or legal guardians. The research team and school teachers informed the parents or legal guardians of participants the day before the survey and had them voluntarily sign an informed consent form. All participants participated in the survey voluntarily. This project was organized by the China Physical Fitness Surveillance Center under the approval of the General Administration of Sports of China. Furthermore, information about children was gathered by teacher interviews, and their consent was obtained. At the beginning of this study, all data were completely anonymized.

### 2.3 Measures

The test indicators of PAB and PH between 3 and 6 years in kindergarten were investigated and tested by NPFMSM [7]. The manual was compiled by the General Administration of Sports of China in 2003. Since its application, it has been investigated in a large number of children aged 3 to 6 years, and can objectively reflect the real-time physical health status of Chinese children with high reliability and validity. In addition, before the start of the survey, the researchers obtained the informed consent of the subjects through paper forms.

#### 2.3.1 Independent variables: Physical activity behavior

Participants’ PAB during kindergarten was investigated based on their physical activity performance. We classified activities by type of activity (DPA, such as taking physical education classes and playing games; SPA, such as painting, reading, and attending cultural classes), location of activity (indoor and outdoor), frequency of activity (days/week), and duration of activity (minutes/day) and divided kindergarten PAB into eight survey indicators: DPA frequency (days/week), dynamic indoor physical activity (DIPA) duration (minutes/day), DOPA frequency (days/week), DOPA duration (minutes/day), static indoor physical activity(SIPA) frequency (days/week),SIPA duration (minutes/day), static outdoor physical activity (SOPA) frequency (days/week), and SOPA duration (minutes/day). We then designed an open-ended self-administered questionnaire. Considering that the participants were too young and did not have the ability to complete the questionnaire independently, this questionnaire was completed by teachers. The questionnaire was explained by a dedicated person with training experience before completion.

#### 2.3.2 Dependent variable: Physical health monitoring index

All test indicators were completed in accordance with the test requirements and test specifications of the NPFMSM. The test values were recorded immediately upon completion of the test and data were entered into the computer twice in a timely manner to avoid data entry errors.

*2*.*3*.*2*.*1*. *Demographic indicators*. Demographic indicators included age and sex. Age was calculated based on the time the test was completed.

*2*.*3*.*2*.*2*. *Body morphology indicators*. Height (cm) and weight (kg) were measured, using the same types of instruments recommended by the NPFMSM [7]. Subjects were required to dress minimally and stand straight, barefoot and at ease when being measured. Weight was measured to the nearest 0.1 kg with a standardized scale, and height was measured to the nearest 0.1 cm with a portable stadiometer. Both the scales and stadiometer were calibrated before use.

*2*.*3*.*2*.*3*. *Physical fitness indicators*. In this study, the physical fitness was mainly used to reflect children’s motor skill level and motor ability, and the PF of children was assessed by a six-indicator test. The tests were the standing long jump (SLJ), tennis throwing (TT), sit-and-reach (SR), 10 m shuttle run test (10-mSRT), balance beam walking (BBW), and double-leg timed hop (DTH), which reflect lower limb strength, upper limb strength, flexibility, speed quality, balance, and agility, respectively. All tests were completed by professionals with training experience. The tests were conducted three times and the best scores were recorded. During the test, all participants were verbally encouraged by the tester to complete the test with maximum effort.

### 2.4 Statistical analyses

The data from the PAB questionnaire and PH monitoring index test in kindergarten for children aged 3 to 6 years were analyzed using IBM SPSS Statistics version 26.0 software. The children’s PH testing indicators were scored on a 5-point scale and classified into 4 levels (excellent, good, pass, fail) according to the evaluation criteria of the NPFMSM. PABs at kindergarten were analyzed in terms of physical activity duration, physical activity type and physical activity place, including eight indicators of TDPA, average duration of physical activity per day (ADD), DPA, DIPA, DOPA, SPA, SIPA and SOPA. The test values of each index were counted and analyzed in terms of time. Descriptive statistics were used to analyze the distribution and trends of PH score and PAB. An independent sample T test was used to compare the gender differences of each test index. A chi-square test was used to analyze the differences in PH levels and physical activity time among children of different ages. The percentile method was used to classify physical activity into three levels, and physical activity time was divided into short, moderate, and long (T1, T2, and T3). Pearson correlation analysis was used to examine the correlation between PH and PAB; linear regression was used to analyze the correlation between PH and PAB, and the significance level was set at p<0.05.

## 3 Results

### 3.1 PH characteristics description

**Fig 1:**
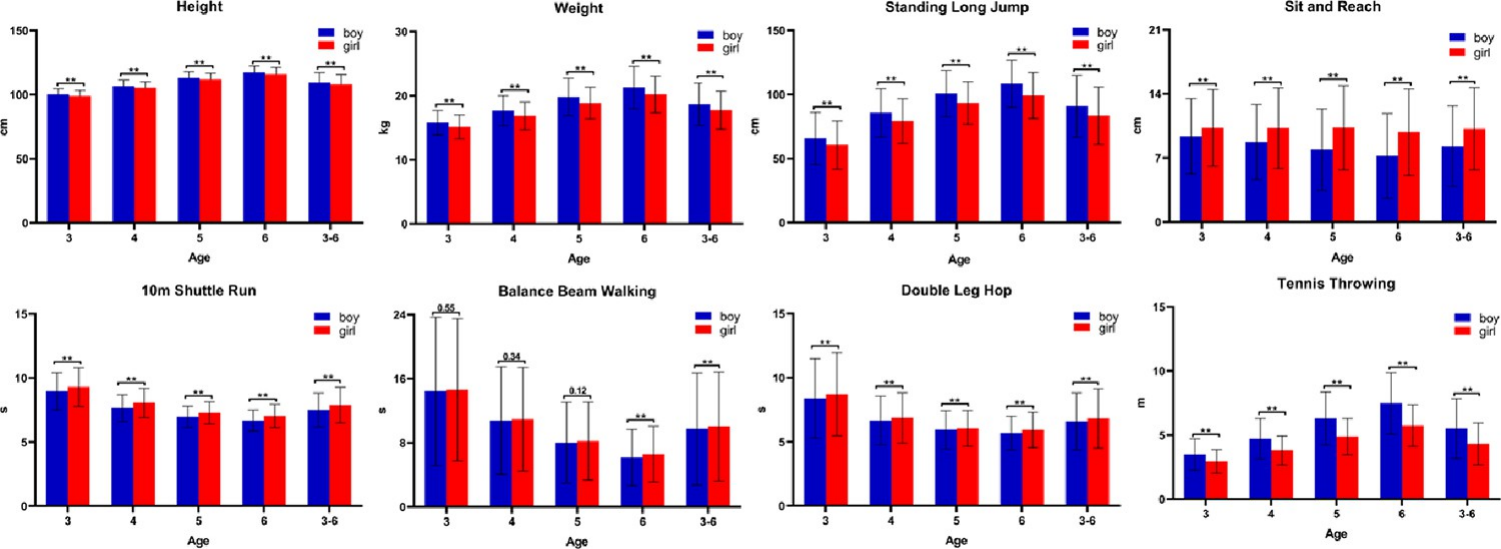
Gender differences in the components of physical health of young children, controlling for age (Blue for boys, Red for girls). The X-axis describes the age and the Y-axis describes the test value. * indicates statistically significant test values for boys versus girls (P<0.05).

Table 1 describes the grade distribution and age incremental differences in PH levels of children by gender. The PH level of young children was most concentrated in L3 (pass), and its probability distribution was 69.38% for the total PH score, 58.48% for the PF score; and 54.39% for the BM score, with a relatively low rate of excellent attainment. The excellent and good rates of the PH and PF scores showed a decreasing trend with increasing age, and BM was relatively stable. The failure rate of each index, on the other hand, showed a uniform characteristic, and both had an increasing trend in age. The level of BM was superior to that of PF among the components of PH. The distribution of PH levels of children in all age groups was significantly different (P < 0.01). Fig 1 shows the differences in the test scores of each element of PH for boys and girls. Except for SR, the test scores of girls’ PH components were lower than those of boys, and most of them had significant differences (P < 0.01),. Only the BBW did not reflect gender differences when aged 3 to 5 years.

**Table 1:** Differences in PH levels of young children with increasing age [N (%)].

Age (N)	PH score (%)						PF score (%)						BM score (%)					
	L1	L2	L3	L4	X^2^	P	L1	L2	L3	L4	X^2^	P	L1	L2	L3	L4	X^2^	P
Boys (9168)					329.64	**<0.01**					398.31	**<0.01**					112.21	**<0.01**
3	30	303	1304	201			16	362	1104	356			133	377	1192	136		
(1838)	(1.6)	(16.5)	(70.9)	(10.9)			(0.9)	(19.7)	(60.1)	(19.4)			(7.2)	(20.5)	(64.9)	(7.4)		
4	29	380	1869	356			18	468	1561	587			204	734	1435	261		
(2634)	(1.1)	(14.4)	(71)	(13.5)			(0.7)	(17.8)	(59.3)	(22.3)			(7.7)	(27.9)	(54.5)	(9.9)		
5	54	440	1985	443			27	560	1664	671			303	805	1480	334		
(2922)	(1.8)	(15.1)	(67.9)	(15.2)			(0.9)	(19.2)	(56.9)	(23)			(10.4)	(27.5)	(50.7)	(11.4)		
6	**7**	107	1153	507			**1**	129	887	757			**138**	509	941	186		
(1774)	**(0.4)**	(6)	(65)	(28.6)			**(0.1)**	(7.3)	(50)	(42.7)			**(7.8)**	(28.7)	(53)	(10.5)		
Girls					60.4	**<0.01**					61.19	**<**0.01				99.74		**<0.01**
(8846)																		
3	23	228	131	305			13	300	1159	397			118	365	1088	298		
(1869)	(1.2)	(12.2)	3(70.3)	(16.3)			(0.7)	(16.1)	(62)	(21.2)			(6.3)	(19.5)	(58.2)	(15.9)		
4	38	359	1761	391			20	489	1494	546			269	536	1322	422		
(2549)	(1.5)	(14.1)	(69.1)	(15.3)			(0.8)	(19.2)	(58.6)	(21.4)			(10.6)	(21)	(51.9)	(16.6)		
5	31	353	1851	557			8	482	1579	723			260	721	1364	447		
(2792)	(1.1)	(12.6)	(66.3)	(19.9)			(0.3)	(17.3)	(56.6)	(25.9)			(9.3)	(25.8)	(48.9)	(16)		
6	4	180	1221	231			3	284	1052	297			108	317	867	344		
(1636)	(0.2)	(11)	(74.6)	(14.1)			(0.2)	(17.4)	(64.3)	(18.2)			(6.6)	(19.4)	(53)	(21)		

The data are presented as frequency and percentage.

The p values in bold indicate significant differences (p<0.05).

PH score: physical health score: PF score: physical fitness score: BM score: body morphology score.

### 3.2 PAB characteristics description

The types of PAB in kindergarten were divided into dynamic physical activity behavior and static physical activity behavior. Table 2 shows the time distribution of the types of PAB in kindergarten for young children. Overall, the distribution of children’s weekly TDPA in kindergarten was 1355.68 minutes for boys and 1344.58 minutes for girls, with the ADD exceeding 4 hours with no gender difference (P > 0.05). There was no significant change in the total time spent in PA with increasing age, but the type of activity changed. There was a decrease in DPA (9.5% decrease) and an increase in SPA (13.8% increase), with the most significant increase in SIPA. The distribution of physical activity time by percentile was divided into T1 (short), T2 (moderate), and T3 (long) levels, and Table 3 shows the age differences for the distribution of PA time by gender. The differences in TDPA between boys and girls were not significant (P > 0.05) at each age level, while the differences in the distribution levels of DPA and SPA were significant at each age level (P < 0.01). The time distribution of different PAB types was significantly different at all ages (P < 0.01), but without sex differences(P>0.05), only at 6 years of age was the difference in the sex distribution of the SPA significant (P < 0.01). In addition, the place of physical activity was correlated with the type of activity, with DPA dominated by outdoor activities and SPA dominated by indoor activities. The differences in the distribution of time spent in indoor versus outdoor activities by the type of activity were significant (p < 0.01).

**Table 2:** Time distribution of PAB types.

Gender	Age	TDPA (min/w)	ADD (min/d)	DPAB			SPAB		
				DPA (min/w)	DOPA (min/w)	DIPA (min/w) ^c^	SPA (min/w) ^b^	SOPA (min/w) ^b^	SIPA (min/w) ^b^^, c^
Boys	3	1356.6 (16.45)	271.32 (3.29)	816.23 (9.32)	483.37 (5.2)	332.87 (6.08)	540.36 (9.66)	132.63 (4.23)	407.74 (7.99)
	4	1353.01 (12.25)	270.6 (2.45)	814.48 (7.16)	482.66 (4.09)	331.83 (4.86)	538.53 (6.85)	163.72 (3.91)	374.81 (5.54)
	5	1360.54 (11.37)	272.11 (2.27)	798.37 (6.24)	484.04 (3.88)	314.33 (3.8)	562.17 (6.56)	166.15 (3.62)	396.02 (5.22)
	6	1350.68 (16.11)	270.14 (3.22)	737.91 (8.98)	447.09 (5.4)	290.82 (5.04)	612.76 (9.92)	135.81 (4.07)	476.96 (8.7)
	Total	1355.68 (6.79)	271.14 (1.36)	794.88 (3.85)	437.39 (5.88)	295.97 (6.06)	560.8 (3.97)	134.81 (4.26)	486.03 (9.06)
Girls	3	1310.52 (15.34)	262.1 (3.07)	798.88 (9.02)	479 (5.08)	319.88 (5.87)	512.81 (8.8)	125.46 (3.91)	387.35 (7.18)
	4	1362.18 (12.54)	272.44 (2.51)	815.66 (7.39)	486.84 (4.17)	328.82 (5.03)	546.52 (6.97)	157.78 (3.86)	388.75 (5.72)
	5	1345.67 (11.82)	269.13 (2.36)	790.71 (6.56)	481.67 (4.06)	309.04 (3.92)	554.96 (6.79)	147.65 (3.49)	407.31 (5.66)
	6	1354.19 (17.54)	270.84 (3.51)	733.36 (10.28)	437.39 (5.88)	295.97 (6.06)	**620.84 (10.26)** ^**a**^	134.81 (4.26)	486.03 (9.06)
	Total	1344.58 (6.93)	268.92 (01.39)	789.02 (4.02)	474.41 (2.34)	314.61 (2.54)	555.8 (3.98)	143.5 (1.94)	412.3 (3.34)

Data are presented as marginal means and standard error.

DPAB: dynamic physical activity behavior; SPAB: dynamic physical activity behavior; TDPA: total duration of physical activity; ADD: average daily duration; DPA: static physical activity time; DOPA: dynamic outdoor physical activity; DIPA, dynamic indoor physical activity; SPA, static physical activity; SOPA: static outdoor physical activity time; SIPA: static indoor physical activity.

Statically significant values are in bold.

a: girls versus boys, p < 0.05

b: DPAB versus SPAB, p < 0.05

c: outdoor physical activity versus indoor physical activity, p < 0.05.

**Table 3:** Differences in PAB of young children with increasing age [N (%)].

Age	TDPA (min/w)					DPA (min/w)					SPA (min/w)				
	T1	T2	T3	X^2^	P	T1	T2	T3	X^2^	P	T1	T2	T3	X^2^	P
Boys (9168)															
3	421 (22.90)	962 (52.30)	455 (24.80)	5.774	0.673	408 (22.20)	971 (52.80)	459 (25.00)	56.582	**<0.01**	390 (21.20)	994 (54.10)	454 (24.70)	47.108	**<0.01**
4	655 (24.90)	1326 (50.30)	653 (24.80)			657 (24.90)	1423 (54.00)	554 (21.00)			641 (24.30)	1344 (51.00)	649 (24.60)		
5	720 (24.60)	1514 (51.80)	688 (23.50)			526 (18.00)	1703 (58.30)	693 (23.70)			577 (19.70)	1738 (59.50)	607 (20.80)		
6	435 (24.50)	934 (52.60)	405 (22.80)			442 (24.90)	925 (52.10)	407 (22.90)			362 (20.40)	969 (54.60)	443 (25.00)		
Girls (8846)															
3	439 (23.50)	981 (52.50)	450 (24.10)	1.172	0.997	422 (22.60)	1014 (54.20)	434 (23.20)	34.080	**<0.01**	466 (24.90)	953 (51.00)	451 (24.10)	56.568	**<0.01**
4	616 (24.20)	1318 (51.70)	615 (24.10)			636 (25.00)	1386 (54.40)	527 (20.70)			585 (23.00)	1328 (52.10)	636 (25.00)		
5	681 (24.40)	1460 (52.30)	651 (23.30)			534 (19.10)	1571 (56.30)	687 (24.60)			568 (20.30)	1683 (60.30)	541 (19.40)		
6	401 (24.50)	844 (51.60)	391 (23.90)			395 (24.10)	874 (53.40)	367 (22.40)			356 (21.80)	891 (54.50)	389 (23.80)		

The data are presented as frequency and percentage.

The p values in bold indicate significant differences (p<0.05).

### 3.3 Analysis of the association between PG and PAB

The correlation analysis showed (as shown in Table 4) that PAB in kindergarten had a high correlation with the PH of children, and the type of PAB had different correlation effects with PH. TDPA and DPA had a significant positive correlation with the PH score and PF score for children between the ages of 3 and 6 years (P < 0.01). SPA showed a significant positive correlation between PH score and PF score for girls (P < 0.01), while it was not statistically significant for boys (P > 0.05). Differentiating between age and type of PAB, TDPA had a statistically significant positive association with the PH score and PF score at 4 and 5 years of age (P < 0.01), but not at 3 and 6 years of age for boys (P > 0.05). DPA had a statistically significant positive association with PH score and PF score at all ages in boys (P < 0.01), but not in girls at ages 3 and 6 (P > 0.05). SPA had a statistically significant positive relationship with PH score and PF score only at age 5 years (P < 0.05) and negatively affected PH and PF at ages 3 and 6 years for boys (r = -0.008/-0.01/-0.005/-0.004). The relationship between all types of PAB and BM was not statistically significant at most ages (p > 0.05).

**Table 4:** Correlation analysis of PAB and PH by gender and age.

Physical activity behavior	Gender	PH score					PF score					BM score				
		3	4	5	6	Total	3	4	5	6	Total	3	4	5	6	Total
TDPA	Boys	0.026	0.089**	0.108**	0.032	0.069**	0.027	0.101**	0.123**	0.04	0.078**	0.009	-0.01	0.048**	0.005	0.015
	Girls	0.039	0.082**	0.102**	0.068**	0.077**	0.022	0.107**	0.116**	0.073**	0.086**	0.095**	-0.005	0.040*	0.005	0.032**
DPA	Boys	0.055*	0.118**	0.152**	0.068**	0.118**	0.053*	0.129**	0.159**	0.075**	0.125**	0.042	-0.015	0.084**	0.023	0.034**
	Girls	0.055*	0.112**	0.146**	0.043	0.098**	0.005	0.045*	0.062**	0.004	0.098**	0.105**	0.003	0.068**	-0.004	0.046**
SPA	Boys	-0.008	0.036	0.042*	-0.01	0.003	-0.005	0.045*	0.062**	-0.004	0.013	-0.025	-0.001	0.004	-0.013	-0.007
	Girls	0.012	0.029	0.037*	0.072**	0.035**	0.006	0.052**	0.055**	0.085**	0.050**	0.059*	-0.013	0.004	0.013	0.009

* At the 0.05 level (two-tailed), the correlation is significant.

** At the 0.01 level (two-tailed), the correlation is significant.

### 3.4 Regression analysis

**Fig 2:**
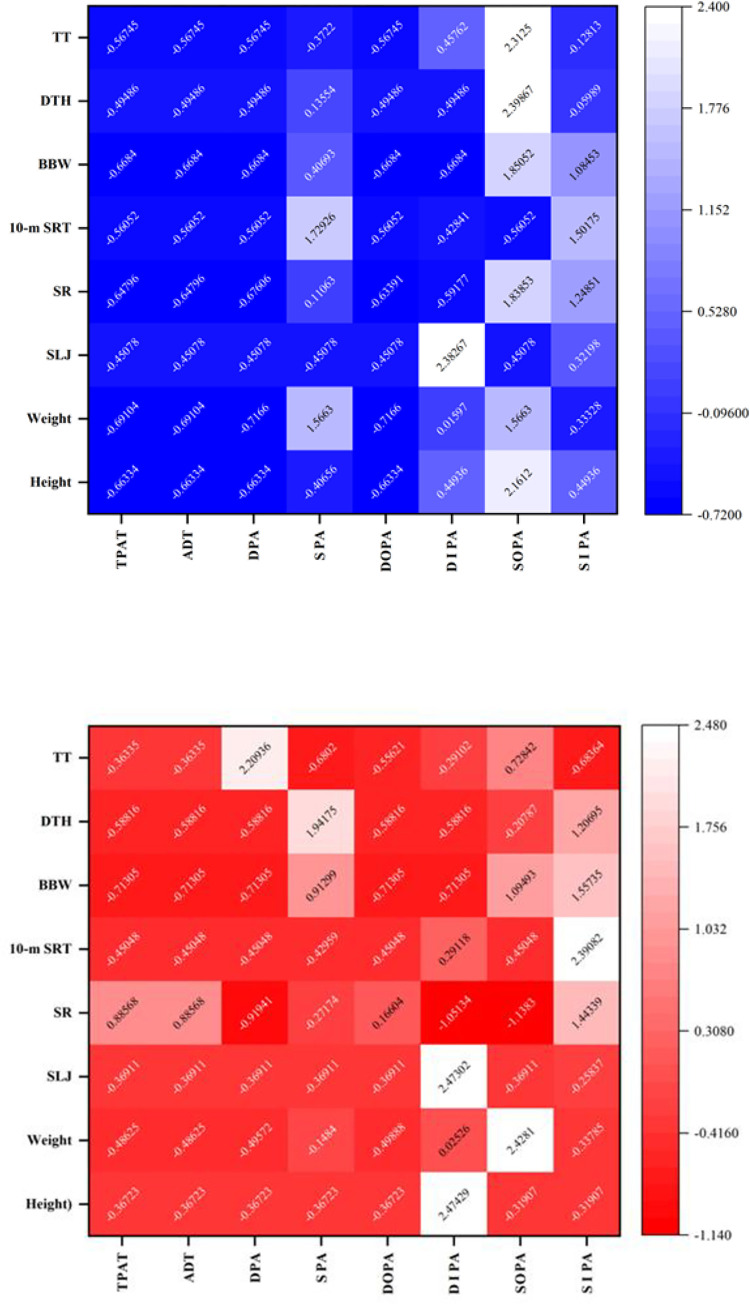
Heat map of the significance coefficients of PH components and PAB time in children, controlling for age, by gender (blue for boys, red for girls).

The regression analysis was conducted separately by gender with PH score, PF score and BM score as dependent variables, and TDPA and all types of physical activity time as independent variables, correcting for age, as shown in Table 5. The effects of PAB on the PH score and PF score of children were mainly from TDPA and DOPA (p = 0). BM scores were only associated with outdoor dynamic physical activity. To further analyze the internal causal factors of PAB in kindergarten on the PH of children, a heatmap of the correlation coefficients between the time spent in various types of PAB and the components of PH was drawn (Fig 2). As seen in the figure, there is a relative consistency in the internal causal factors of the effect of PAB on PH. Static activities have a poor positive contribution to the PH of children and even produce a negative correlation, and some indoor dynamic activities have no effect on PH factors. The correlations between PAB and PH for boys, were mainly found in (1) ST and 10-mSRT and weight; (2) indoor dynamic activity and SLJ; (3) outdoor static activity and height, weight, SR, BBW, DTH and TT; and (4) indoor static activity and height, SLJ, SR, 10-mSRT and BBW, which were not statistically significant. The correlations between PAB and PH for girls were mainly found in (1) DPA and TT; (2) SPA and BBW and DTH; (3) DIPA and height and SLJ; (4) SOPA and body weight, BBW and TT; and (5) SIPA and SR, 10-mSRT, BBW and DTH (P >0.05).

**Table 5:** Independent association between PH and PAB, controlling for age, by gender.

	PH score						PF score						BM score					
	Boys			Girls			Boys			Girls			Boys			Girls		
	R^2^	β	P	R^2^	β	P	R^2^	β	P	R^2^	β	P	R^2^	β	P	R^2^	β	P
TDPA	0.005	0.069	**0**	0.006	0.077	**0**	0.039	0.078	**0**	0.007	0.086	**0**	0.001	0.015	0.146	0.001	0.032	0.003
**DT**	0.048			0.019			0.051			0.019			0.002			0.003		
Outdoor		0.132	**0**		0.143	**0**		0.134	**0**		0.145	**0**		0.043	**0**		4.666	**0**
Indoor		0.014	0.628		-0.023	0.044		0.006	0.56		-0.022	0.056		0	0.986		0.142	0
ST	0.031			0.002			0.033			0.003			0.001			0		
Outdoor		0.014	0.179		0.029	0.006		2.187	0.029		0.036	0.001		-0.016	0.132		0.006	0.567
Indoor		0.008	0.449		0.024	0.022		1.287	0.198		0.036	0.001		0.001	0.894		0.008	0.430

The data are presented as standardized regression coefficient, and interval variables were entered into their original values.

The p values in bold indicate statistical significance for the corresponding predictor in the model with the physical health (p<0.05).

## 4 Discussion

Because the PH of young children is related to their growth, development and health, it is crucial to investigate the factors influencing PH and to adopt appropriate interventions for PH promotion. The results of this study indicate that PAB in kindergarten affects the development of PH in young children. The main findings were that TDPA and DOPA were the primary factors affecting children’s PH; DOPA was the indicator with the best effect on children’s PH, which was positively correlated with all elements of children’s PH, and the overall effect relationship was DOPA > DPA > TDPA > ADD > DIT > SPA > SOPA >SIPA.

### 4.1 PH of young children

Young children in this study performed poorly in terms of PH, with lower PF scores than BM scores. The overall level of PF of young children was concentrated in the passing grade, with low rates of excellent and good attainment and showed a decreasing trend with increasing age, while the failure rate showed the opposite trend. This is basically consistent with the results of the China Early Childhood Physical Health Monitoring Report [8, 9]. The gender differences in the components of PH were clear, and the test scores of boys were generally better than those of girls, except for SR. Only flexibility was higher in girls than in boys during early childhood.

### 4.2 PAB of young children in kindergarten

The children’s PAB in kindergarten included both dynamic and static types of activities, distributed indoors and outdoors. The ADD accumulated during the children’s stay in kindergarten exceeded 4 hours. In the early years, dynamic physical activity behavior was the main form of PAB in kindergarten, and was concentrated outdoors. As age increases, there is a shift to static indoor activities. There was no significant difference in the distribution of total time spent in physical activity among children at each age, but differences in the distribution of time levels under the increment were demonstrated in different activity styles. The impact of the shift in physical activity style is an important factor in the physical fitness of young children.

### 4.3 Physical activity and PH

Numerous studies have confirmed the role of physical activity in promoting PH in young children. Aadland [10] confirmed from a metabolic health perspective that different levels of physical activity are strongly associated with metabolic health in young children, and the degree of this association varies from strong to weak depending on the level of physical activity. Raistenskis J [11] concluded that young children’s PH is associated with the duration of daily moderate-to-vigorous physical activity. Not surprisingly, the dosage relationship and targeting effects of physical activity on PH in young children have been previously explored from different perspectives, similar to the results of our study, which concluded that there is an association between physical activity and PH level, and further analyzed the association between PH and PAB in kindergarten.

### 4.4 Dynamic and static physical activity behavior and PH

Correlation analysis showed that TDPA was strongly related to the total PH score and PF score, both of which showed positive correlation. This indicates that appropriate physical activity during kindergarten has a positive effect on the PH of young children. The effect on BM showed positive correlations only at the ages of 3 and 5 years, which were not significant. Distinguishing the types of PAB, dynamic activity behaviors include game, playing and attending physical education classes. Static behaviors are those with metabolic equivalents (METs)≤6.276 kJ/(kg-h), such as learning and recreation in a sitting, leaning or lying position, except for sleeping [12–14].

The two PAB types produced different effects on PF levels. Regression analysis showed that dynamic activity had a positive effect on all aspects of PF, while static activity performed poorly and even had a negative effect. The decrease in dynamic activity and increase in static activity with increasing age is favorable evidence that static activity affects the decrease in fitness levels. Tremblay [15] argued that static activity in young children exceeding 120 (min/d) caused problems in health status, self-esteem, and decreased academic performance. In addition, some studies have suggested that static physical activity time is negatively correlated with basic motor skills, and most of the physical fitness test indicators in this study were skill-based, further confirming the negative effect of static activity behaviors on young children’s PH [13].

### 4.5 Indoor and outdoor physical activity behavior and PH

In terms of the place of PA, outdoor activities are more closely related to young children’s PH, especially dynamic activities performed outdoors, which positively contribute to all elements of young children’s physical fitness. Compared with outdoor activities, indoor exercise time had significant time-dependent features, and the increase in indoor activity time for older children was an important reason for the lowest level of physical fitness excellence in all. Leigh M [16] concluded that outdoor activities are more conducive to the accumulation of moderate-to-high intensity physical activity. In contrast, indoor activities restrict physical activity due to space, lighting and air circulation, making it difficult to develop a positive effect on PH. Our study found that indoor physical activity did not correlate with several indicators of PH, with slight differences between boys and girls. Only dynamic indoor activities were strongly correlated with some of the test indicators, such as SLJ and 10-mSRT for boys and height and SLJ for girls. The effects of both outdoor and indoor static activities on PH in our study did not reach significance, but they were closely related to individual test indicators.

### 4.6 Limitations and strengths of the study

Due to objective constraints, surveys of children’s physical activity time were completed by teachers as proxies, and values were taken as the average of a week of PA, which may bias the calculation of children’s physical activity time. Future studies should use objective measurement tools to adjust the results of the study to further prove the relationship between PH and PAB in kindergarten. In addition, the study did not conduct a dose-effect analysis of different PAB and PH and its components, and it is particularly important to standardize the time spent on diverse types of physical activity to better support the promotional effect of PAB on PH.

## 5 Conclusion

Our study mainly explored the relationship between PH and PAB in children aged 3–6 years in kindergarten, and concluded that PAB in kindergarten has a positive promoting effect on PH. It is suggested that kindergartens should arrange a reasonable duration of physical activity for children based on the type and place of physical activity. The kindergartens should also appropriately increase dynamic outdoor physical activities and reduce static exercises to improve children’s PH.

## Supporting information

S1 File (DOCX)

S1 Data (XLS)
