# Supplementary material for: Association between physical health and physical activity behaviors for children aged 3–6 years in kindergarten: A cross-sectional study from China
Source: PLoS One. 2022 Dec 15;17(12):e0278341. doi: 10.1371/journal.pone.0278341 (PMC9754297; doi:10.1371/journal.pone.0278341)
Supplement: S1 File — (DOCX) [file pone.0278341.s001.docx]

Informed Consent Form

Dear parents:

We sincerely invite your child to participate in a study on the association between physical activity behaviors and physical health of children aged 3 to 6. This study has been reviewed and approved by the ethics committee of the Department of psychology of Beijing Normal University (approval No: 201903260036). This informed consent form is to seek your consent. It will introduce you to the purpose, steps, benefits, risks, inconvenience and your rights and interests of the study. Please carefully read it and carefully decide whether to participate in the study. When the researcher explains and discusses the informed consent form to you, you can ask questions at any time and ask him/her to explain what you don't understand. You can discuss with your family and your teacher before making a decision.

**1. Research background and purpose**

Physical activity is closely related to human health. Adequate and regular physical activity is an important cornerstone of children’s physical and mental health, effectively promoting children’s bone health and cardiometabolic health, improving cardiopulmonary fitness and physical fitness, promoting cognitive function development and helping to control weight and reduce the risk of psychological depression . With the rapid development of modern society, human lifestyles and behavioral habits are undergoing major changes. The sedentary lifestyles of screen viewing, traveling by car and sitting in the office are becoming more common and occurring at a younger age. The World Health Organization (WHO) has ranked sedentary behavior as the fourth highest risk factor for mortality worldwide. According to statistics, approximately 61% to 80% of young children worldwide spend too much time in static activity, and the proportion in China is as high as 88.2%, which is much higher than the global average . Children's PH problems caused by insufficient physical activity have become an important public health issue facing the current society.

Objective: To explore associations between physical activity behavior in kindergarten and physical fitness of children aged 3-6 years , and provide scientific basis for improving children's physical activity behavior and health.

**2. Research Introduction**

2.1. Experimental time

The main work of this experiment is to collect children's physical activity behavior and physical health data.

1) You will undergo the following checks to determine whether you can participate in the study:

① Ask and record your medical history and treatment history, and conduct physical examination;

② It is necessary to screen for sports system diseases, including inflammation or injury of joints or muscles, which seriously affect the movement of the body, etc.

2) After you participate in the screening test, if you meet the criteria for joining the study, you will receive the following tests and surveys:

① The questionnaire of sleep duration shall be filled in by parents and teachers;

② Body morphology test: including height and weight;

③ Motor ability test: including tanding long jump, sit-and-reach, 10-m shuttle run test, balance beam walking, double-leg timed hop, tennis throwing.

3) After you complete the above examination, we will provide you with a report on physical activity behavior and physical health

**3. Inclusion and exclusion criteria**

3.1. Inclusion criteria

1) School children aged 3 to 6;

2) With the consent of parents and teachers, there are no diseases that seriously hinder movement after screening;

3) Those who voluntarily participate in the experiment and can participate in the whole process, and sign the informed consent form.

3.2. Exclusion criteria

1) Before the test, patients with heart, liver, spleen, kidney and other major organs or other potential safety diseases and a history of motion syncope were detected, such as:

① Respiratory diseases, tracheitis, asthma, lung diseases, etc;

② Cardiovascular and cerebrovascular diseases, arrhythmia, heart disease, hypertension, etc;

③ Circulatory system diseases: diabetes, gastroenteritis, etc;

④ Dynamic system disease: inflammation or injury of joints or muscles, which seriously affects the movement of the body, etc.

2) Subjects who stayed up late and fasting before motor ability test.

3) In case of extreme discomfort such as chest tightness and poor breathing during the test, stop the test immediately and contact the school hospital, emergency center, test teachers or relevant staff.

Note: doctors and parents should be informed about the prevention of hidden diseases they do not know. For those who have recently suffered from acute diseases such as high fever and diarrhea, those who have not recovered their physical strength such as staying up late continuously, and girls who are in the physiological cycle, please pay attention to their own special reactions or accidents in the motor ability test. You can explain to the test teacher or researcher that if you can't adhere to it, you can slow down or avoid the test.

**4. What will you need to do if you participate in the study?**

1) Before you are enrolled in the study, the researcher will ask and record your medical history, and conduct relevant physical examination, body composition, motor system diseases and other examinations. You are a qualified participant. You can voluntarily participate in the study and sign the informed consent form. If you are unwilling to participate in this study, we will give other appropriate treatment according to your condition and wishes.

2) If you volunteer to participate in the study, you will follow the following steps:

This study will be divided into two parts to complete the experiment. The questionnaire was explained by the researcher and filled in by parents and teachers; The test part is organized by researchers under the guidance of teachers. It mainly includes the test of body shape and physical quality.

**5. Possible adverse reactions, discomfort and risks of participating in the study**

In this test, you will test in class. The exercise intensity starts from low intensity and gradually increases. We will terminate the test in time according to your fatigue signs, heart rate, or possible symptoms. During the test, there may be abnormal blood pressure, dizziness, tachycardia, bradycardia or arrhythmia, as well as rare cases such as heart disease, stroke and death. When you feel tired or other discomfort, it is important to ask to stop the test.

**6. Possible adverse reactions, risks, discomfort and inconvenience of participating in the study**

Most of the tests in this study were completed by the subjects at rest without adverse reactions and other risks. The motor ability test process will be fully demonstrated with teachers, and its content will be designed as a teaching plan. After the test is completed in teaching, teachers can monitor the whole test process to avoid sports risk accidents. This kind of risk is mainly reflected in dizziness, fatigue and other symptoms caused by excessive exercise load, which can be restored to the original state by taking a few minutes after exercise.

**7. Confidentiality**

All information about you, including your identity, medical history, condition, physical examination and laboratory examination results, will be kept strictly confidential to the extent permitted by law. Subject related researchers are allowed to check your medical records related to this study to confirm the authenticity and accuracy of the data collected in this study, but do not involve your personal details. Your name will not appear in any public information or report related to this study.

**8. Your rights**

Your participation in the research is entirely voluntary. You have the right to withdraw from the research at any stage of the experimental research, and your withdrawal from the research will not be subject to any punishment or loss of interest, nor will it affect the teachers' teaching to you. If you decide not to participate in the study and withdraw from the study at any time after the start of the study, please contact the researcher in time. The investigator may terminate the study without your consent if:

1) Out of consideration of your sports risk;

2) As a subject, you failed to comply with the relevant provisions of the study, failed to participate in sports classes as required or failed to carry out various examinations and tests on time;

3) Study termination.

Signature:

Date:
